# True prevalence of twin HDV-HBV infection in Pakistan: a molecular approach

**DOI:** 10.1186/1743-422X-8-420

**Published:** 2011-09-04

**Authors:** Asad U Khan, Muhammad Waqar, Madiha Akram, Mehnaz Zaib, Muhammad Wasim, Shahzad Ahmad, Zeeshan Niaz, Sajid Ali, Haider Ali, Muhammad Idrees, Mohammad A Bajwa

**Affiliations:** 1 Department of Microbiology, Hazara University Mansehra, Pakistan; 2 Genome Center for Molecular Diagnostics & Research, 945 J-II Johar Town Lahore, Pakistan; 3 Khyber Medical University, Hayatabad Road, Peshawar, Khyber Pakhtoonkhaw, Pakistan; 4 Division of Molecular Virology, CEMB, University of the Punjab Lahore-53700, Pakistan; 5 Department of Gastroenterology, Sheikh Zayed Medical Complex, Lahore, Pakistan

## Abstract

Hepatitis Delta Virus (HDV) infects only patients that are already infected by hepatitis B virus (HBV) because this is sub satellite virus which depends on and propagate only in the presence of HBV. HDV causes co-infection or super infection with sever complication as compared to only HBV infection. No study on molecular level on HDV is available from this region; therefore, the aim of this study was to found out the molecular epidemiology of HDV (as a co-infection with HBV) in different geographical regions of Pakistan.

Total 228 HBsAg positive samples were received for the study from different geographical regions of the country. Only HBV DNA PCR positive samples were further utilized for the presence of HDV RNA. For this purpose, HDV RNA and HBV DNA was extracted and amplified using reverse transcriptase polymerase chain reaction (RT-PCR), nested PCR and real-time PCR.

Out of the total 228 HBsAg positive samples, HBV DNA was detected in total 190 (83.3%) samples belonged to different patients. Of these 190 patients, HDV RNA was observed in 53 (28%) patients. Of the 53 HDV positive cases, 37 (69.8%) were males and 16 (30.2%) were female patients. The percentage of dual infection was found higher significantly (p < 0.05) in male patients as compared to female patients. Total 41 (26.8%) patients were below 40 years and 13 (31.7%) were above 40 years of age. No significant difference was seen in patients with ages above or below 40 years. In the provinces of Sindh, Khyber Pakhtoonkhaw and Punjab the observed prevalence of HDV was 67%, 6% and 4% respectively.

In conclusion, the HDV infection is not uncommon in Pakistan and its prevalence is higher significantly in the Province of Sindh (p < 0.01) and male six (p < 0.05).

## Introduction

Hepatitis Delta virus (HDV) infection is present globally and infects human being already infected by Hepatitis B virus (HBV). Hepatitis Delta is mostly present in Africa; South America, Romania, Russia and the Mediterranean region included Southern Italy [1]. HDV was first discovered by Rizzetto in the patients that were already infected by HBV in year 1980 [2]. HDV is a sub-satellite virus associated with HBV to cause severe acute and chronic forms of liver infection [3]. Tthe particle size of HDV is about 36-nm that require hepatitis B surface antigen (HBsAg) for their enveloped and transmission [4]. The HDV genome is a circular, negative sense, single-strand RNA, which is approximately 1700 nucleotides in length [5]. These nucleotides is assemble with two viral proteins HD Ag-S and HDAg-L to form a ribonucleoprotein [6]. These Two Hepatitis D Ag proteins are translated from viral mRNA and this process is called RNA editing [7]. The dual infection of HBV and HDV occurs in the form of co-infection or as a super-infection. The Super infection of HDV with HBV caused a progressive chronic liver disease up to (80%), which further enhances liver cirrhosis and hepatocellular carcinoma (HCC). Co-infection by both HBV and HDV viruses causes more severe acute liver disease and is a higher risk for the development of fulminate hepatitis compared to only HBV infected patients [8].

It has been estimated that approximately 5% of HBV carriers are co-infected with HDV, leading to an estimated 15 million persons infected with HDV worldwide [9]. HDV infection has a worldwide distribution, but its frequency varies greatly throughout different geographic regions. It is highly endemic in the Middle East, in the Mediterranean area, in the Amazonian region, and in several African countries [10].

Several studies propose that most people acquired HDV infections through parenteral and sexual routes [11-13]. Furthermore the reported seroprevalence of HDV infection among HBV carriers is significantly higher in intravenous drug users (IDU) compared to the non-IDU population [14].

In Pakistan, the prevalence of chronic HBV infection is estimated to be 16-57% in general population predominantly in younger males living in rural areas [15,16]. However, in these studies enzyme linked immunosorbant assay (ELISA) based assays were utilized for the estimation of this prevalence rate. For the true prevalence of HDV-HBV co-infection, we used molecular based methods such as PCR and real-time PCR for the detection of HBV DNA and HDV RNA in HBsAg positive samples from patients' different geographical regions of Pakistan.

## Methods

### Source of Clinical Samples

A total of 228 HBsAg positive serum samples by enzyme linked immunosorbant assay (ELISA) were received at Genome Centre for Molecular Based Diagnostics & Research (GCMBDR) Lahore, Pakistan from different geographical regions of the country for HBV DNA and/or HDV RNA detection from January 2011 to March 2011. GCMBDR is a state of the art molecular based diagnostic facility that provides general public sensitive, specific and more reliable diagnostic tests on cost-to-cost basis utilizing PCR and real-time PCR methods. All sera were stored in aliquots at -70°C till was used for nucleic acid (DNA/RNA) isolation. Demographic characteristics, serological and biochemical data was available for 190 patients (male 121; female 69, mean age 42 years [range, 6-82 ± SD] years)) with chronic HBV infection and were thus included in the data analysis. These samples belonged to different provinces/geographical regions of Pakistan. Seventy were from Sindh, 66 belonged to Khyber Pakhtoonkhaw (KPK) and 54 were from Punjab. There was no need for separate written informed consent from subjects for this study, since this analysis was a part of the original protocol in a routine workup of Molecular Diagnostics.

### HBV DNA extraction and real-time PCR amplification

Qualitative and quantitative detection of serum HBV DNA in all patients were performed by SmartCycler II real-time PCR (Cepheid, USA) with an internal RNA standard derived from the 5' UTR. The utilized kits for HBV DNA extraction and real-time PCR were done according to the procedures given in the kit protocols (Sacace Biotechnologies Italy). Sensitivity of the assay is 20 IU per ml blood sample. Specificity of the assay is about 99%.

### Extraction of HDV RNA and complementary DNA (cDNA) synthesis

HDV RNA was extracted from 100 μL serum sample using Gentra RNA isolation kit (Life Technologies, USA). About 50 ng of the extracted RNA was reverse transcribed into cDNA with Molony-murin leukemia virus reverse transcriptase enzyme (Gibco BRL, Life Technologies USA). The thermal cycling was carried out in thermal cycler for 50 minutes at 37°C. The reaction mixture for the preparation of cDNA, for a single reaction contained 8 50 mM Tris-HCl (pH 8.3), 7.5 mM KCl, 3 mM MgCl2, 0.1 M DTT, 10 mM dNTPs and 200 U of MMLV reverse transcriptase enzyme. At the end of reaction, MMLV was heat inactivated at 95°C for 5 minutes.

### Qualitative detection

The detection of the HDV cDNA was carried using nested PCR. The Qualitative detection of hepatitis Delta virus was done in two rounds. First round PCR was carried out in a PCR reaction tube that contained 8 μL of mix I which having 2.5 mM MgCl2, 100 μM concentration of each of the four deoxynucleotides, 10 pM of each outer sense nucleotides 695-718 {5'-CATGGTCCCAGCCTCCTCGCTGGC-3'} and outer anti-sense primers; nucleotides 873-896 {5'- CCGCGAGGAGGTGGAGATGCCATG-3'} [17], and 1 U of Taq DNA polymerase Enzyme. In the thermal cycler, firstly the samples were incubated on 95°C for 2 min then it was followed by 35 cycles consisting of 95°C for 1 min, 64°C for 1 min and 72°C for 1 min. The second round (nested PCR) PCR was also carried out in a reaction tube that contained 8 μL of mix II which had 2.5 mM MgCl2, a 100 μM concentration of each of the four deoxynucleotides, 10 pM of each inner sense nucleotides 729-748 (5'-CAACATTCCGAGGGGA CCGT-3') and inner anti-sense primers nucleotides 846-865 (5'-GAAGGAAGGCCCTCGAGAACAAGA-3') [17]. For standard PCR to seen contamination we used the negative and reagent controls along with samples in each run. Finally the PCR products were electrophoresis on a 2% agarose gel prepared in 1× Tris-borate-EDTA (TBE) buffer, stained with ethidium bromide, and evaluated under UV transilluminator. The sizes of PCR products were estimated according to the migration pattern of a 50-bp DNA ladder (Fermentas Life Sciences). The sensitivity limit of this qualitative detection method was 10 copies per ml blood sample.

### Statistical Analysis

All the data was analyzed and the summary statistic was carried out by SPSS version 10.0 (a statistical package). Variables are given in the form of rates (%). Chi Square test was used for categorical variables that measured association among categorical variables. Values of less than 0.05 were considered significant

## Results

**Figure 1:**
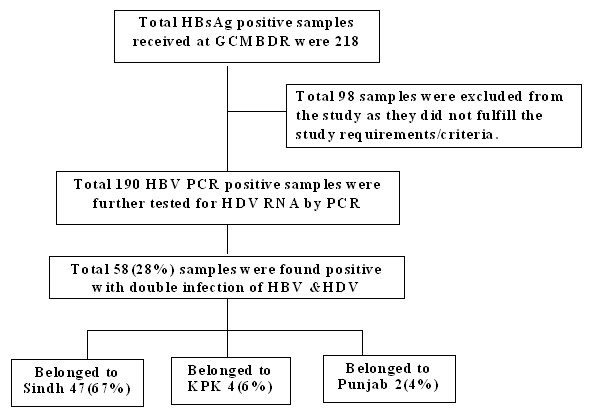
***Study disposition and enrolments***. Total 288 consecutive HBsAg positive patient's samples were received at Genome Centre for Molecular based Diagnostics & Research. Of these total 98 patients samples were excluded from the study either the volume of sera were not sufficient for testing (n = 19) or failed to meet inclusion criteria of the study (n = 79) as they were HBV DNA negative by real-time PCR. Total 190 patients with chronic HBV who fulfilled the study criteria were enrolled for this study Out of 190 enrolled patients, 63.7% (n = 121) were males and 36.3% (n = 69) were females. Total 58(28%) samples were found positive with double infection of HBV &HDV. In the provinces of Sindh, Khyber Pakhtoonkhaw (KPK) and Punjab the observed prevalence of HDV was 67%, 6% and 4% respectively.

The study enrolments and patients' disposition are shown in Figure 1. One hundred and ninety patients with chronic HBV who fulfilled the study criteria were enrolled for this study out of total 288 consecutive HBsAg positive patients. Total 98 patients samples were excluded from the study either the volume of sera were not sufficient for testing (n = 19) or failed to meet inclusion criteria of the study (n = 79) as they were HBV DNA negative by real-time PCR. Out of 190 enrolled patients, 63.7% (n = 121) were males and 36.3% (n = 69) were females. The mean age was 38 ± 11 years. The sensitivity of the assay was excellent that is 20 IU per ml blood sample.

Of the total 190 tested HBV positive cases, HDV RNA was detected in 58 (28%) patients. Out the HDV positive patients, 37 were males and 16 were females. The rates of HDV positivity in both sexes are shown in table 1. The HDV RNA data was also analysed Province wise (Table 2). In the provinces of Sindh, Khyber Pakhtoonkhaw (KPK) and Punjab the observed prevalence of HDV was 67%, 6% and 4% respectively. The co-infection of HDV with HBV was higher significantly in the Province of Sindh as compared to Provinces of KPK and Punjab. No significant difference was seen in the rates of HBV+HDV co-infection in patients below or above 40 years of ages (Table 3).

**Table 1:** Rate of HBV + HDV co-infection in male and female patients

S.N	Gender	Total samples HBV positive	Found positive with HDV	Percentage
1	Male	121	37	31%

2	Female	69	16	23%

	**Total**	**190**	**53**	**28%**

**Table 2:** Rate of HBV + HDV co-infection in different provinces of Pakistan

S.N	province	Total HBV DNA PCR positive isolates	Found positive with HDV	Percentage
1	SINDH	70	47	67%

2	KHYBER PUKHTUN KHWA	66	4	6%

3	PUNJAB	54	2	4%

	**Total**	**190**	**53**	**28%**

**Table 3:** Rate of HBV + HDV co-infection in different patients age groups

S.N	Age group	Total HBV DNA PCR positive isolates	Found positive with HDV	Percentage
1	Below 40Y	149	40	26.8%

2	40Y & above	41	13	31.7%

	**Total**	**190**	**53**	**27.9%**

## Discussion

HDV can be caused either as a co-infection (concomitant) in persons infected with HBV or as a super-infection (subsequent) in chronic HBV carriers. As it is now an established fact that HDV is a defective virus, containing particles of RNA nucleoprotein in virion-like form, present in patients with acute hepatitis B and chronic hepatitis that requires the presence of a hepadnavirus (HBV) for full replication. That is why for its penetration into the hepatocytes and assembly of virion, it needs the help of HBV that provides the viral coat surface antigen and can survive only in the presence of HBV (co-infection or/and super-infection). Here we report on the HDV-HBV co-infection or molecular epidemiology of HBV+HDV co-infection. Our study is the first one to describe the molecular epidemiology of HDV and its co-infection with HBV in different geographical regions of Pakistan. This is very important study as it describes the dual HBV-HDV infection using molecular methods. It has already been reported that in contrast to HBV mono-infection, HBV+HDV co-infection leads to exacerbation and rapid progression of chronic liver disease, hepatic failure, and deaths in patients [18,19].

Several important findings emerged from the results of this study. The first finding of the current study showed that more than 83% of the HBsAg positive patients by ELISA were also positive by HBV DNA PCR. HBV+HDV co-infection was observed in 28% HBV DNA positive patients that is very alarming. This is the true prevalence of active HDV infection in HBV DNA positive cases. The previously conducted studies on the seroprevalence of HDV infection in Pakistan have reported the prevalence of antibodies against HDV (HDV-Ab) of 16% and 57%, respectively [15,16]. However these studies were based on ELISA methods that might give both false positive and false negative results. In spite of this high prevalence, no data on molecular epidemiology of HDV in Pakistan are available. The prevalence of HDV positive cases obtained in this study is extremely high. More recently a high endemicity of HDV in Cameroonian populations was reported by Foupouapouognigni and colleagues [20].

Next important finding of the study is the observation that HDV rate is significantly high (p < 0.001) in male (69.81%) as compared to female (30.18%) patients. However the chances of this co-infection is same in all age groups as no significant difference was seen for the rates of co-infection in patients with ages below or above 40 years. This finding of the current study is contrary to the finding of another study from Pakistan where HDV was predominant in young population [15].

Another important finding of the present study is the observation that rates of HDV is significantly high (P < 0.001) in ethnic group Sindhi as compared to Punjabi and Pashtoon races. Is Sindhi racing an independent risk factor for acquiring HDV, is question marked? To correctly answer this question, we recommend further conformation of this observation by other studies with large number of patients. In our study in all three groups the baseline characteristics of patients were similar however we were unable to establish the exact mode of contamination that may play a major role in this high rate of HDV in Sindh. No study on correlation of HDV-HBV co-infection with ethnic group is available from this region.

Also so far no epidemiological survey at molecular level has been documented in Pakistan to investigate the prevalence of double HBV plus HDV hepatitis infection hence this study provides new insights and dimensions for the molecular virologists to carry out investigations on patients having this double hepatitis related liver disorders. Further we believe that the results of the current study enrich the limited information on co-infection and molecular epidemiology of HDV in the region.

## Conclusion

Our study showed a higher prevalence of HDV in HBV DNA positive patients that highlights the importance of screening for HDV infection in HBV carriers. Its prevalence is higher significantly in the Province of Sindh and male six. Clinicians particularly gastroenterologist, hepatologists and other medical and practitioners should be made aware of the danger of twin infection with HDV and HBV. Further studies, with full genome characterization of different HDV strains are required.

## Competing interests

The authors declare that they have no competing interests.

## Authors' contributions

AUK and MW conceived the study. AUK and MW collected the samples and performed PCRs and other the molecular analysis. MA and MZ helped in molecular analysis. MW, SA, ZN, SA, HN and MAB helped in literature search and drafting manuscript. MI critically reviewed the manuscript and revised it. All the authors read and approved the final manuscript.
